# Long-term outcome of patients with adolescent idiopathic scoliosis seeking nonoperative treatment after a mean follow-up of 42 years

**DOI:** 10.1007/s43390-022-00541-5

**Published:** 2022-07-11

**Authors:** Mazda Farshad, Tobias Götschi, David E. Bauer, Thomas Böni, Christoph J. Laux, Method Kabelitz

**Affiliations:** 1 Department of Orthopedics, Balgrist University Hospital, University of Zurich, Forchstrasse 340, 8008 Zurich, Switzerland; 2 Institute for Biomechanics, ETH Zurich, 8008 Zurich, Switzerland

**Keywords:** Adolescent idiopathic scoliosis, Bracing, Nonoperative treatment, Long-term results, Spinal deformity

## Abstract

**Purpose:**

Adolescent idiopathic scoliosis (AIS) affects up to 3% of otherwise healthy adolescents. The extreme long-term outcomes of nonoperative treatment are underreported. This study aimed to investigate the long-term outcome of nonoperative-treated AIS patients. Comparison between a bracing and an observation approach were performed.

**Methods:**

In a retrospective cohort study, 20 nonoperatively treated AIS patients were observed concerning patient-related outcome measures (PROM) (visual analog scale (VAS), Short Form Health Survey 36 item (SF 36), Scoliosis Research Society (SRS 24), Oswestry Low Back Pain Disability Index (ODI), Psychological General Well-Being Index (PGWBI)), radiological curve progression and health-related quality of life (HRQoL). Baseline characteristics and radiological imaging were collected. At follow-up, anteroposterior and lateral X-rays as well as questionnaires were analyzed.

**Results:**

Twenty patients (16 females, mean age: 14.6 ± 3.2 years) with a follow-up time of 42 ± 9 years were included. Nine patients (initial Cobb 35° ± 19°) were treated with bracing for a mean time of 26 ± 9 months, while the other 11 patients (initial Cobb 29° ± 11°) were observed. The primary curve progressed from 32° ± 15° to 52° ± 25° in average with no significant difference between the cohorts (*p* = 0.371). At final follow-up, a mean ODI score of 7 ± 7.9 points with no difference depending on the treatment (*p* = 0.668) was seen. No significant differences were observed for PROMs. Curve magnitude correlated neither at diagnosis (*p* = 0.617) nor at follow-up (*p* = 0.535) with the ODI score at final follow-up.

**Conclusion:**

After a mean of 42 years, patients with nonoperative treatment of moderate AIS demonstrated a good clinical outcome despite progression of the deformity.

**Level of evidence:**

Level IV, therapeutic study.

## Introduction

Adolescent idiopathic scoliosis (AIS) is a three-dimensional structural deformity of the spine developing during adolescence after the age of 10 years [1]. The etiopathogenesis of AIS is considered multifactorial and largely remains unclear [2]. The prevalence of AIS, defined as a coronal curvature with a Cobb’s angle greater than 10°, is about 3% in healthy adolescents, mostly affecting female patients [3]. Even though typically asymptomatic during adolescence, AIS curves can progress and may cause back pain, shortness of breath, incapacity in conducting activities of daily living, psychological impairment and esthetic concerns in adulthood [4–6]. The choice of treatment depends on the magnitude of the primary curve, curve progression, patient age and symptoms, such as back pain and cardiac or respiratory compromises [7].

Operative treatment is traditionally reserved for adolescent patients with major curves exceeding 45° to stabilize the curves and prevent late progression and deterioration of cardio-pulmonary symptoms and health-related quality of life (HRQoL) [8]. Recent data show clinically relevant improvements in functional and health-related quality of life, self-image, and pain at the 2-year follow-up after fusion surgery for AIS, with only few adverse events [9]. Particularly in view of the good short-term surgical results in terms of patient-reported outcomes today, the question arises as to long-term treatment recommendations and the future value of nonoperative therapy.

Patients presenting a curve magnitude of ≥ 25° or a rapid curve progression (> 5° in 6 months) with bony immaturity (Risser sign < 3) and pre-menarchal status are subject to bracing therapy [10–12]. However, evidence on the influence of wearing time, patients’ age, curve flexibility or skeletal maturity on the results of bracing after a very long time is underreported [11, 13]. If untreated, one-third (32%) of patients with Risser 0–1 experience curve progression resulting in psychological complications [6]. In addition to the radiological and functional long-term outcome of nonoperative AIS treatment, the current research is focused on quality of life and patient satisfaction [14, 15]. However, increasing life expectancy is shifting interest to extreme long-term outcomes of different therapeutic modalities.

While there is long-term data on AIS published, most cohorts focus on clinical or patient-reported outcome data and do not always provide radiographic follow-up. The aim of this study was to present the extreme long-term outcome of patients with AIS treated with either bracing or observation regarding radiographic curve progression, associated pain, disability, and health-related quality of life (HRQOL).

## Materials and methods

### Study population

**Fig. 1:**
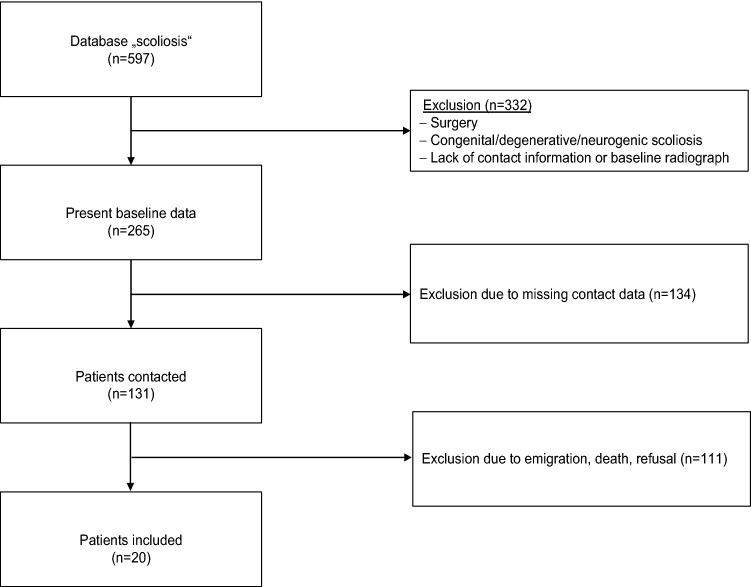
Patient acquisition process

Patient files in the form of paper copies in the archive of our institution were searched for the diagnosis of “scoliosis”. Between 1960 and 1990, 597 patients with degenerative, congenital, neurogenic, or operatively treated scoliosis were treated in the outpatient clinic of our institution. Patients with AIS with a minimum Cobb angle of 10° in the major curve, age of 10–20 years at the time of diagnosis as well as nonoperative treatment, availability of a full standing anteroposterior X-ray of the spine at diagnosis and a minimum follow-up of 20 years were subject to this analysis. Accordingly, 332 patients not meeting the inclusion criteria were excluded from this study. From the remaining 265 patients, contact information could be obtained in 131 cases. These patients were contacted via mail or phone. However, 111 of these patients were excluded due to various reasons (e.g., emigration, refusal for participation, death, or nonresponse). Finally, a total of 20 patients could be included (Fig. 1). During follow-up, asymptomatic patients without relevant curve progression (> 5° per 6 months) were asked to present again whenever they had symptoms.

### Radiological examination

Plain film radiographs at diagnosis were digitized for measurement of radiographic parameters. Follow-up anteroposterior and lateral radiographs were performed with a microdose 3D imaging system (EOS™ imaging, SA, 10 rue de Mercoeur, 75011 Paris, France). The magnitude of the primary and secondary curve was measured using Cobb’s method. Radiological imaging was analyzed using IMPAX (version 6.4.0.6010) and IMPAX Orthopaedic Tools (Agfa-Gevaert N.V., Mortsel, Belgium).

### Questionnaires

At the follow-up visit, patients were asked about their medical history and socio-demographic as well as disease-specific data. Further, the EQ-5D-5L, Short Form Health Survey 36 item (SF 36), Scoliosis Research Society (SRS 24), Oswestry Low Back Pain Disability Index (ODI) and Psychological General Well-Being Index (PGWBI) were recorded [16–21]. In addition, we asked all patients to remember their pain levels during adolescence and to rate them on the visual analog scale (VAS).

### Ethics approval

The study was approved by the local ethics committee prior to patient enrollment and a signed informed consent was obtained from each patient. Patient data were retrieved from the local hardcopy archive and the electronic database. Study data were collected and managed using REDCap electronic data capture tools hosted at our institution for analysis [22].

### Statistics

Patient characteristics are presented with mean and standard deviation or median with range, as applicable. Predictor–outcome associations were assessed using Spearman rank correlation tests. Differences between the groups with/without brace were compared using the Mann–Whitney *U* test. Data analysis was conducted with SPSS (IBM SPSS Statistics for Windows, Version 25.0. Armonk, NY: IBM Corp.). *p* values below 0.05 were considered statistically significant.

## Results

Twenty patients (80% female) with a mean age at diagnosis of 14.6 ± 3.2 years agreed to undergo follow-up examination in adulthood. Nine patients (45%) were treated with bracing therapy over an average time of 26 ± 16 months. The remaining 11 patients were treated without bracing. No crossover from the observation to the bracing cohort was documented. The follow-up periods were 29 years to a maximum of 58.8 years in the brace group and 33.4 years to a maximum of 55.3 years in the observation group, respectively. There was no statistical difference between the investigated cohorts regarding age, BMI, length of follow-up, pain at diagnosis or major curve Cobb angle at diagnosis (Table 1). During the entire time of follow-up, no patient underwent spinal surgery, neither corrective nor due to degenerative changes.

**Table 1:** Patients’ characteristics

	All patients (*n* = 20)	Brace cohort (*n* = 9)	Observation cohort (*n* = 11)	*p* value
Sex				
Female	16 (80%)	9 (100%)	7 (63.6%)	
Male	4 (20%)	0	4 (36.4%)	
Age (yr) at diagnosis	14.6 (± 3.2)	13.5 (± 3.2)	15.5 (± 3.1)	0.07
Age (yr) at final follow-up	56.5 (± 7.8)	57.3 (± 9.6)	55.9 (± 6.5)	0.91
Follow-up (yr)	42 (± 9)	43.3 (± 11.1)	41 (± 7.4)	0.77
Duration of bracing (months)		26.1 (± 15.6)		
Body mass index at follow-up	23.2 (± 4)	24.1 (± 5.4)	22.5 (± 2.4)	0.77
Pain (VAS) at diagnosis (median, minimum; maximum)	1 (0; 7)	0 (0; 7)	0 (0; 6)	0.74
Major curve Cobb angle (°) at diagnosis	31.7 (14.6)	35 (± 18.6)	28.9 (± 10.5)	0.75

Results, if not stated differently, in mean (± standard deviation). yr, year; VAS, visual analog scale

Significance defined as *p* < .05

### Radiologic findings

Baseline and follow-up anteroposterior whole spine radiographs were available for all included patients. During the observation period, the major curve progressed from 32° ± 15° to 52° ± 25° on average. Individuals without brace treatment had an average increase from 29° ± 11° to 45° ± 21°, whereas braced patients developed from 35° ± 19° to 59° ± 28° in their major curve. Secondary curves evolved in average by 10° ± 12° in unbraced and 12° ± 18° in braced patients. Tables 2 and 3, respectively, shows the individual patients' baseline and follow-up measurements including patient-reported outcome measures of both patient cohorts. There were no differences in major curve progression between the two cohorts (*p* = 0.568) and between sexes (*p* = 0.91).

**Table 2:** Baseline and follow-up measurements and PROMs of patients with observation

Patient										EQ-5D-5L						PGWBI	SRS-24				SF-36							
	Gender	Duration follow-up (yr)	VAS diagnosis	Curve magnitude diagnosis (°)	Curve magnitude follow-up (°)	Difference curve magnitude (°)	Working ability	ODI	VAS back	Mobility	Self-care	Usual activities	Pain	Anxiety/Depression	VAS		Pain	Self-image	Function	Activity	Physical functioning	Physical role	Bodily pain	General health	Vitality	Social functioning	Emotional role	Mental health
Observation																												
1	f	36.0	0	30	39	9	1	16	4	2	1	2	2	2	68	64	4.5	5	3.7	4.7	80	100	62	32	50	75	67	60
2	m	54.3	0	38	84	46	4	0	0	1	1	1	2	1	85	101	3.7	4.7	4.3	4.7	100	100	100	67	85	100	100	96
3	m	41.5	6	15	23	8	1	0	1	1	1	1	1	1	95	104	4	4.3	5	5	100	100	100	92	85	100	100	96
4	f	35.3	3	45	52	7	1	20	7	2	1	3	3	1	65	50	4.8	4.3	3.3	3.3	80	0	22	55	25	88	33	56
5	f	42.7	0	26	52	26	1	8	2	1	1	1	1	1	90	94	3.8	4.7	4	4.7	95	100	62	87	80	100	100	92
6	m	33.4	0	29	65	36	1	0	0	1	1	1	1	1	90	95	3.5	4.7	4.3	5	100	100	100	97	75	100	100	84
7	f	37.8	4	20	28	8	1	2	1	1	1	1	1	1	100	91	3.7	5	4.3	5	100	100	84	87	75	100	100	76
8	m	35.1	0	14	15	1	1	0	0	1	1	1	1	1	90	86	3.8	5	4.3	5	100	100	100	87	65	100	100	72
9	f	55.3	0	37	64	27	4	2	0	1	1	1	1	1	80	103	3.7	5	4.3	5	95	75	100	77	80	100	100	100
10	f	41.7	0	41	45	4	2	16	3	1	1	1	1	1	90	93	4.2	4.7	4	4.7	85	100	74	92	70	100	100	84
11	f	38.5	0	23	30	7	1	4	0	1	1	1	2	1	80	78	4.5	5	4.3	4.7	90	100	62	57	55	88	100	68

### Disability and back pain

At final follow-up, the mean ODI score was 7 ± 7.9 points. The cohort treated with bracing showed a mean ODI of 8.5 ± 8.6 points compared to 6.2 ± 7.3 points in the observation group (*p* = 0.668). There was no correlation between ODI at follow-up and the magnitude of the major curve at the time of diagnosis (*p* = 0.617) or at follow-up (*p* = 0.535). In addition, there was no correlation between the ODI at follow-up and the age at diagnosis (*p* = 0.771).

At the time of final examination, 15 patients (75%) showed a working ability of 100%. Among the others, one person was unable to work due to back pain. Four patients were already retired.

The median VAS for pain was 1 (range 0–7), which was consistent until follow-up without differences among the braced (1, range 0–5) and observed (1, range 0–7)) patients. Also, subcategories for pain in the EQ-5D, SF-36, SRS-24 and ODI questionnaire did not show statistically significant differences between both groups.

### Health-related quality of life

Considering the EQ-5D-5L, patients showed no or minor limitations in their mobility (median: 1 (IQR 0 versus median 1 (IQR 0), *p* = 0.842), self-care (1 (IQR 0) vs 1 (IQR 0), *p* = 1.000) or daily activities (1 (IQR 0) vs 1 (IQR 0.5), *p* = 0.882) (80%) at final follow-up. There was a slight, but statistically insignificant difference in pain with a median of 2 points (IQR 1.5) in the braced group compared to 1 point (IQR 1) in the observed group (*p* = 0.412).

The SRS-24 questionnaire generally yielded “good” (4 out of 5) to “very good” (5 out of 5) scores for all subcategories as shown in Tables 2 and 3, respectively. There was no subcategory showing statistically significant differences in scores when comparing the two cohorts. Some categories were more favorable for the observation group (e.g., “going out” 3.2 ± 0.6 vs. 2.8 ± 1.1 or “daily activities” 4.6 ± 1.2 vs. 4.1 ± 1.8), whereas two subcategories concerning pain showed a slight tendency to increased pain symptoms (“maximum pain” 3.4 ± 2.1 vs. 3 ± 1.7 and “everyday pain” 2.4 ± 1.5 vs. 2.2 ± 1.3). No patient reported to regularly require medication against back pain.

**Table 3:** Baseline and follow-up measurements and PROMs of patients with bracing

Patient										EQ-5D-5L						PGWBI	SRS-24				SF-36							
	Gender	Duration follow-up (yr)	VAS diagnosis	Curve magnitude diagnosis (°)	Curve magnitude follow-up (°)	Difference curve magnitude (°)	Working ability	ODI	VAS back	Mobility	Self-care	Usual activities	Pain	Anxiety/Depression	VAS		Pain	Self-image	Function	Activity	Physical functioning	Physical role	Bodily pain	General health	Vitality	Social functioning	Emotional role	Mental health
Bracing																												
12	f	38.1	0	28	47	19	1	0	1	1	1	1	1	1	95	96	3.8	2.3	4.3	5	100	100	100	92	80	100	100	84
13	f	29.0	6	27	24	− 3	1	16	3	1	1	1	2	1	90	65	4.2	5.7	3.3	3.7	95	75	51	92	70	100	100	84
14	f	35.4	0	31	57	26	1	12	2	1	1	3	3	3	90	78	4.2	4	5	4.7	80	100	62	97	80	88	100	68
15	f	41.0	0	26	29	3	1	10	2	1	1	1	2	1	98	97	3.8	4.7	4	4.7	85	100	72	92	70	100	100	88
16	f	57.8	3	26	35	9	4	10	0	1	1	1	1	1	100	108	2.8	5.3	4.3	4.7	85	100	100	90	90	100	100	100
17	f	29.9	0	73	78	5	1	0	0	1	1	1	1	1	90	87	3.7	4	4.3	5	100	100	100	82	60	100	100	72
18	f	49.5	0	23	79	56	1	0	0	1	1	1	1	1	95	95	3.7	4	3.7	5	100	100	100	87	65	100	100	76
19	f	56.3	7	61	109	48	1	2	1	1	1	1	2	1	100	41	3.8	6.3	4	3.7	95	100	74	97	75	100	100	68
20	f	52.3	0	20	75	55	4	27	5	2	1	2	3	1	60	88	4.5	4	4	3	60	50	51	32	75	100	100	76

Yr, year; VAS, visual analog scale; ODI, Oswestry Disability Index; EQ-5D-5L, European Quality of Life 5 Dimensions 5 Levels; PGWBI, Psychological General Well-Being

Index, SRS-24, Scoliosis Research Society 24-item questionnaire; SF-36, Short Form-36 questionnaire

At the time of final follow-up, 80% of the overall cohort were ranked in the “being positive” subcategory of the PGWBI with a mean score of 86 ± 18 out of 110. Ten percent showed “moderate psychological distress” (scores 61–72) and 10% were classified as “experiencing severe psychological distress” (scores 0–60). No difference between the two observed groups could be identified (bracing 84 ± 20, observation 87 ± 20).

The results of the Short Form 36 survey are presented in Table 4 showing no statistical differences between cohorts for all subcategories.

**Table 4:** Mean scores (± SD) of the SF-36 questionnaire subcategories

	All participants (*n* = 20)	Bracing cohort (*n* = 9)	Observation cohort (*n* = 11)	*p* value
Physical functioning	91 (± 11)	89 (± 13)	93 (± 8)	0.552
Role physical	90 (± 25)	92 (± 18)	89 (± 30)	0.941
Body pain	79 (± 23)	79 (± 22)	79 (± 25)	0.882
General health	80 (± 20)	85 (± 20)	76 (± 20)	0.175
Vitality	71 (± 15)	74 (± 9)	68 (± 18)	0.71
Social functioning	97 (± 7)	99 (± 4)	96 (± 8)	0.552
Role emotional	95 (± 16)	100 (± 0)	91 (± 22)	0.503
Mental health	80 (± 13)	80 (± 11)	80 (± 15)	0.882

Significance defined as *p* < 0.05

## Discussion

We aimed to investigate extreme long-term follow-up study of nonoperatively treated patients diagnosed with mild or moderate AIS reporting radiological outcome and level of disability, pain and health-related quality of life after a minimum of 29 years and up to a maximum of 58.8 years of follow-up.

Starting with mild to moderate curves, the major curves progressed by 20° ± 20° within 42 ± 9 years. Patients treated with brace demonstrated a 24° ± 23° increase in curve magnitude, while those without brace treatment had an increase of 16° ± 15°. This is well explainable, as decision for brace treatment was made in younger patients with more potential for curve progression. On extrapolating the results of similar studies with a shorter follow-up time, they were found to coincide with the results of the present study with regard to curve progression [23]. We, however, do not want to claim that brace treatment is ineffective, and we remind the limitations due to age differences at first diagnosis within the selected groups. Larger prospective randomized studies have demonstrated the efficacy of bracing in patients with AIS and bracing should be considered a valuable option in the treatment of mild or moderate AIS [10].

Due to incomplete radiologic imaging of the iliac crests, Risser’s staging at diagnosis could not be consistently obtained for this study. Presumably, Risser stages were not routinely determined at this time due to validation only occurring in 1988 [24].

No patient underwent corrective surgery during the observation period, even though some had extensive scoliotic deformities (curve magnitude: maximum: 109° in the brace group and 84° in the observation group). Nevertheless surprisingly, no patient underwent any kind of spine surgery due to secondary degenerative changes. An interesting observation is that all patients did not return to an orthopedic institution over the long course of their illness, although some patients showed a considerable curve progression. In female AIS patients, pregnancy—including the effect of the peptide hormone relaxin—is a known risk factor for a peripartum curve progression, especially considering the long observation period [25]. In our predominantly female study population, however, there was no significant difference in curve progression between both sexes.

This study has profound limitations. Since pain intensity was not ranked at the time of diagnosis, patients were asked to retrospectively remember their pain levels as accurately as possible, which likely leads to recall bias. The data on pain at diagnosis, therefore, must be interpreted cautiously.

The retrospective nature of the study itself, but also the long enrollment period back to 1960 with significant advancements in spinal deformity surgery, results in additional limitations. We were not able to consistently determine the frequency of follow-up and the reason for its discontinuation. However, it can be assumed that they were suspended only in the absence of curve progression and symptoms. Also, the high proportion of non-included patients of around 85% generates an unquantifiable selection bias.

We observed good clinical outcomes (EQ-5D-5L, PGWBI, SRS-24) after a mean of 42 years despite progression of primary and secondary curves. ODI scores of the observation group demonstrated comparable levels of randomly selected non-scoliotic control groups reported in the literature [26]. As confirmed by other studies, nonoperatively treated AIS patients do not show pronounced symptoms in the long term [4]. In a retrospective case–control study by Danielson et al., patients showed a slightly worse back function (ODI 9.2) 22 years after brace treatment compared to a non-scoliotic control group (ODI 4.8) without influence on the general health-related quality of life. In addition, though showing significantly more degenerative disc changes than the controls, no correlation between pain and its localization and curve size or an increase of 10° or more since end of treatment could be found [26]. Another study, recently conducted by Watanabe et al., showed similar results after a mean follow-up of 25 years (range 12–39) for nonoperatively treated (bracing and/or observation) AIS [27]. In 107 subjects, no significant difference was verified concerning ODI and three out of four domains (pain, function, mental health) of the SRS-22 questionnaire compared to a control group. Such and other reports, including our current report, raise the question whether differences of treatment options are clinically less relevant than expected or if the measures that are used to find clinically relevant differences are not adequate enough [28]. Although it is known that younger patients with AIS may develop greater deformities with potentially more severe secondary degenerative changes, we did not find an association of age at diagnosis with long-term poor back function (*p* = 0.771) in our collective [29].

Keeping the above-mentioned limitations in mind, we conclude that AIS patients undergoing nonoperative treatment of mild to moderate AIS reported good clinical outcomes despite progression of the scoliotic deformity after 29–58.8 years.

## Conclusion

After a mean of 42 years, patients with a desire for nonoperative treatment of mild to moderate AIS demonstrated a good clinical outcome despite substantial progression of the scoliotic deformity.

## References

[1] Asher MA, Burton DC (2006). Adolescent idiopathic scoliosis: natural history and long term treatment effects. Scoliosis.

[2] Wajchenberg M, Astur N, Kanas M, Martins DE (2016). Adolescent idiopathic scoliosis: current concepts on neurological and muscular etiologies. Scoliosis Spinal Disord.

[3] Weinstein SL, Dolan LA, Cheng JC, Danielsson A, Morcuende JA (2008). Adolescent idiopathic scoliosis. Lancet.

[4] Hawes MC, Weinstein SL (2003). Health and Function of Patients with Untreated Idiopathic Scoliosis [2] (multiple letters). J Am Med Assoc.

[5] Erwin J, Carlson BB, Bunch J, Jackson RS, Burton D (2020). Impact of unoperated adolescent idiopathic scoliosis in adulthood: a 10-year analysis. Spine Deform.

[6] Weinstein SL (2019). The natural history of adolescent idiopathic scoliosis. J Pediatr Orthop.

[7] Lonstein JE (2006). Scoliosis. Clin Orthop Relat Res.

[8] Danielsson AJ, Wiklund I, Pehrsson K, Nachemson AL (2001). Health-related quality of life in patients with adolescent idiopathic scoliosis: a matched follow-up at least 20 years after treatment with brace or surgery. Eur Spine J.

[9] Mens RH, Bisseling P, de Kleuver M, van Hooff ML (2022). Relevant impact of surgery on quality of life for adolescent idiopathic scoliosis: a registry-based two-year follow-up cohort study. Bone Jt J.

[10] Weinstein SL, Dolan LA, Wright JG, Dobbs MB (2013). Effects of bracing in adolescents with idiopathic scoliosis. N Engl J Med.

[11] Zhang Y, Li X (2019). Treatment of bracing for adolescent idiopathic scoliosis patients: a meta-analysis. Eur Spine J.

[12] Johnson MA, Gohel S, Flynn JM, Anari JB, Cahill PJ, Winell JJ, Baldwin KD (2022). “Will I Need a Brace?”: likelihood of curve progression to bracing range in adolescent idiopathic scoliosis. Spine Deform.

[13] Dolan LA, Donzelli S, Zaina F, Weinstein SL, Negrini S (2020). Adolescent idiopathic scoliosis bracing success is influenced by time in brace: comparative effectiveness analysis of BrAIST and ISICO cohorts. Spine (Phila Pa 1976).

[14] Simony A, Hansen EJ, Carreon LY, Christensen SB, Andersen MO (2015). Health-related quality-of-life in adolescent idiopathic scoliosis patients 25 years after treatment. Scoliosis.

[15] Watanabe K, Ohashi M, Hirano T, Katsumi K, Mizouchi T, Tashi H, Minato K, Hasegawa K, Endo N (2020). Health-related quality of life in nonoperated patients with adolescent idiopathic scoliosis in the middle years: a mean 25-year follow-up study. Spine (Phila Pa 1976).

[16] EuroQol Group (1990). EuroQol—a new facility for the measurement of health-related quality of life. Health Policy (New York).

[17] Ware JE, Sherbourne CD (1992). The MOS 36-item short-form health survey (SF-36). I. Conceptual framework and item selection. Med Care.

[18] Asher MA, Min Lai S, Burton DC (2000). Further development and validation of the Scoliosis Research Society (SRS) Outcomes Instrument. Spine (Phila Pa 1976).

[19] Fairbank JC, Couper J, Davies JB, O’Brien JP (1980). The Oswestry low back pain disability questionnaire. Physiotherapy.

[20] Bullinger M, Heinisch M, Ludwig M (1990). Skalen der Erfassung des Wohlbefindens: Psychometrische Analysen zum „Profile of Mood States“ (POMS) und zum „Psychological General Wellbeing Index“ (PGWI). Z Differ Diagnostische Psychol.

[21] Dupuy H (1984) The Psychological General Well-being (PGWB)Index. In Assessment of Quality of Life in Clinical Trials of CardiovascularTherapies Edited by: Wenger NK, Mattson ME, Furburg CD, Elinson J.New York. Le Jacq Publishing. 170–83

[22] Harris PA, Taylor R, Thielke R, Payne J, Gonzalez N, Conde JG (2009). Research electronic data capture (REDCap)-a metadata-driven methodology and workflow process for providing translational research informatics support. J Biomed Inform.

[23] Haefeli M, Elfering A, Kilian R, Min K, Boos N (2006). Nonoperative treatment for adolescent idiopathic scoliosis: a 10- to 60-year follow-up with special reference to health-related quality of life. Spine (Phila Pa 1976).

[24] Goldberg MS, Poitras B, Mayo NE, Labelle H, Bourassa R, Cloutier R (1988). Observer variation in assessing spinal curvature and skeletal development in adolescent idiopathic scoliosis. Spine (Phila Pa 1976).

[25] Dewan MC, Mummareddy N, Bonfield C (2018). The influence of pregnancy on women with adolescent idiopathic scoliosis. Eur Spine J.

[26] Danielsson AJ, Nachemson AL (2003). Back pain and function 22 years after brace treatment for adolescent idiopathic scoliosis: a case-control study—part I. Spine (Phila Pa 1976).

[27] Watanabe K, Ohashi M, Hirano T, Katsumi K, Mizouchi T, Tashi H, Minato K, Hasegawa K, Endo N (2020). Health-related quality of life in nonoperated patients with adolescent idiopathic scoliosis in the middle years: a mean 25-year follow-up study. Spine (Phila Pa 1976).

[28] Farshad M, Kutschke L, Laux CJ, Kabelitz M, Schüpbach R, Böni T, Jentzsch T (2020). Extreme long-term outcome of operatively versus conservatively treated patients with adolescent idiopathic scoliosis. Eur Spine J.

[29] Akazawa T, Watanabe K, Matsumoto M, Tsuji T, Kawakami N, Kotani T, Sakuma T, Yamamoto T, Demura S, Orita S, Fujimoto K, Shiga Y, Niki H (2018). Modic changes and disc degeneration in adolescent idiopathic scoliosis patients who reach middle age without surgery: can residual deformity cause lumbar spine degeneration?. J Orthop Sci.

